# Klotho antagonizes pulmonary fibrosis through suppressing pulmonary fibroblasts activation, migration, and extracellular matrix production: a therapeutic implication for idiopathic pulmonary fibrosis

**DOI:** 10.18632/aging.102978

**Published:** 2020-04-03

**Authors:** Qiqing Huang, Yan Chen, Shaoran Shen, Yuanyuan Wang, Liya Liu, Shuangshuang Wu, Wei Xu, Weihong Zhao, Mingyan Lin, Jianqing Wu

**Affiliations:** 1 Key Laboratory of Geriatrics of Jiangsu Province, Department of Geriatrics, The First Affiliated Hospital of Nanjing Medical University, Nanjing 210029, Jiangsu, China; 2 Department of Neurobiology, School of Basic Medical Sciences, Nanjing Medical University, Nanjing 211166, Jiangsu, China

**Keywords:** idiopathic pulmonary fibrosis (IPF), klotho (KL), weighted gene co-expression network analysis (WGCNA), precision-cut lung slices (PCLSs), pulmonary fibroblasts

## Abstract

Idiopathic pulmonary fibrosis (IPF) has been widely accepted as an aging-related fatal lung disease with a therapeutic impasse, largely a consequence of the complex and polygenic gene architecture underlying the molecular pathology of IPF. Here, by conducting an integrative network analysis on the largest IPF case-control RNA-seq dataset to date, we attributed the systems-level alteration in IPF to disruptions in a handful of biological processes including cell migration, transforming growth factor-β (TGF-β) signaling and extracellular matrix (ECM), and identified klotho (*KL*), a typical anti-aging molecule, as a potential master regulator of those disease-relevant processes. Following experiments showed reduced *Kl* in isolated pulmonary fibroblasts from bleomycin-exposed mice, and demonstrated that recombinant KL effectively mitigated pulmonary fibrosis in an *ex vivo* model and alleviated TGF-β-induced pulmonary fibroblasts activation, migration, and ECM production *in vitro*, which was partially ascribed to *FOXF1* and *CAV1*, two highly co-expressed genes of *KL* in the IPF. Overall, *KL* appears to be a vital regulator during pulmonary fibrosis. Given that administration of exogenous KL is a feasible treatment strategy, our work highlighted a promising target gene that could be easily manipulated, leaving the field well placed to further explore the therapeutic potential of *KL* for IPF.

## INTRODUCTION

Idiopathic pulmonary fibrosis (IPF) is a chronic, progressive, and ultimately fatal lung disease of unknown etiology, with a median survival time of only 2-4 years after diagnosis [1–3] and limited therapeutic options [4]. As a quintessential aging-related lung disease [5, 6], IPF occurs in middle-aged and elderly adults [7] and its prevalence rises remarkably with age [8]. There is an emerging body of evidence suggesting that anti-aging treatment is a novel therapeutic approach for IPF [9].

KL has long been characterized as an anti-aging molecule with both membrane bound and soluble forms [10], whose deficiency renders multiple organs, such as kidney [11], heart [12], and lung [13], more susceptible to fibrosis, a common feature of aging [14]. Previously, the declines of KL in serum from IPF patients [13] as well as in several mouse pulmonary fibrosis models [13, 15, 16] imply the involvement of KL in the pathogenesis of pulmonary fibrosis. Further clues of the anti-fibrotic potential of KL in the lung include *Kl*-heterozygous hypomorphic allele mice displayed exacerbated asbestos- [16] and bleomycin- [13] induced pulmonary fibrosis, and that both recombinant KL (rKL) supplementation and *Kl*-overexpression attenuated pulmonary fibrosis response. Despite multiple efforts have been made to evaluate the effect of KL on the reverse of hallmarks of aging lung [8], such as cellular senescence [17], mitochondrial dysfunction [16], and stem cells exhaustion [18], the molecular mechanism underlying the protective effect of KL against pulmonary fibrosis remains murky, especially *KL*’s regulatory role in pulmonary fibroblasts, the effector cells during the development of IPF [19].

Therefore, with the rationale that co-expressed genes have a tendency to be functional related, we carried out Weighted Gene Co-expression network analysis (WGCNA) to elucidate the functional role of *KL* on a public RNA-Seq dataset comprising the largest sample size of IPF patients to our knowledge [20]. We reduced the complex and polygenic gene architecture underlying the predisposition to IPF into two functional disease-associated gene modules, in one of which *KL* plays a pivotal role through interacting with genes related to cell migration, TGF-β signaling, and ECM.

To validate our bioinformatic findings, we experimentally assessed the anti-fibrotic effect of *KL* in an *ex vivo* pulmonary fibrosis model and clearly demonstrated the amelioration of fibrosis following rKL supplementation, which was accompanied with the alleviation of pulmonary fibroblasts activation, migration, and ECM production. We further enhanced the validity of our prediction by showing two genes predicted to be functionally related with *KL* could effectively mediate its activity against pulmonary fibrosis. Together, these observations provide new insights into the mechanisms underpinning the anti-fibrotic effect of *KL* and advance our understanding of IPF etiology and potential therapeutics.

## RESULTS

### Integrative network analysis prioritizes *KL* as the top IPF-relevant gene from the systems-level perspective

**Figure 1:**
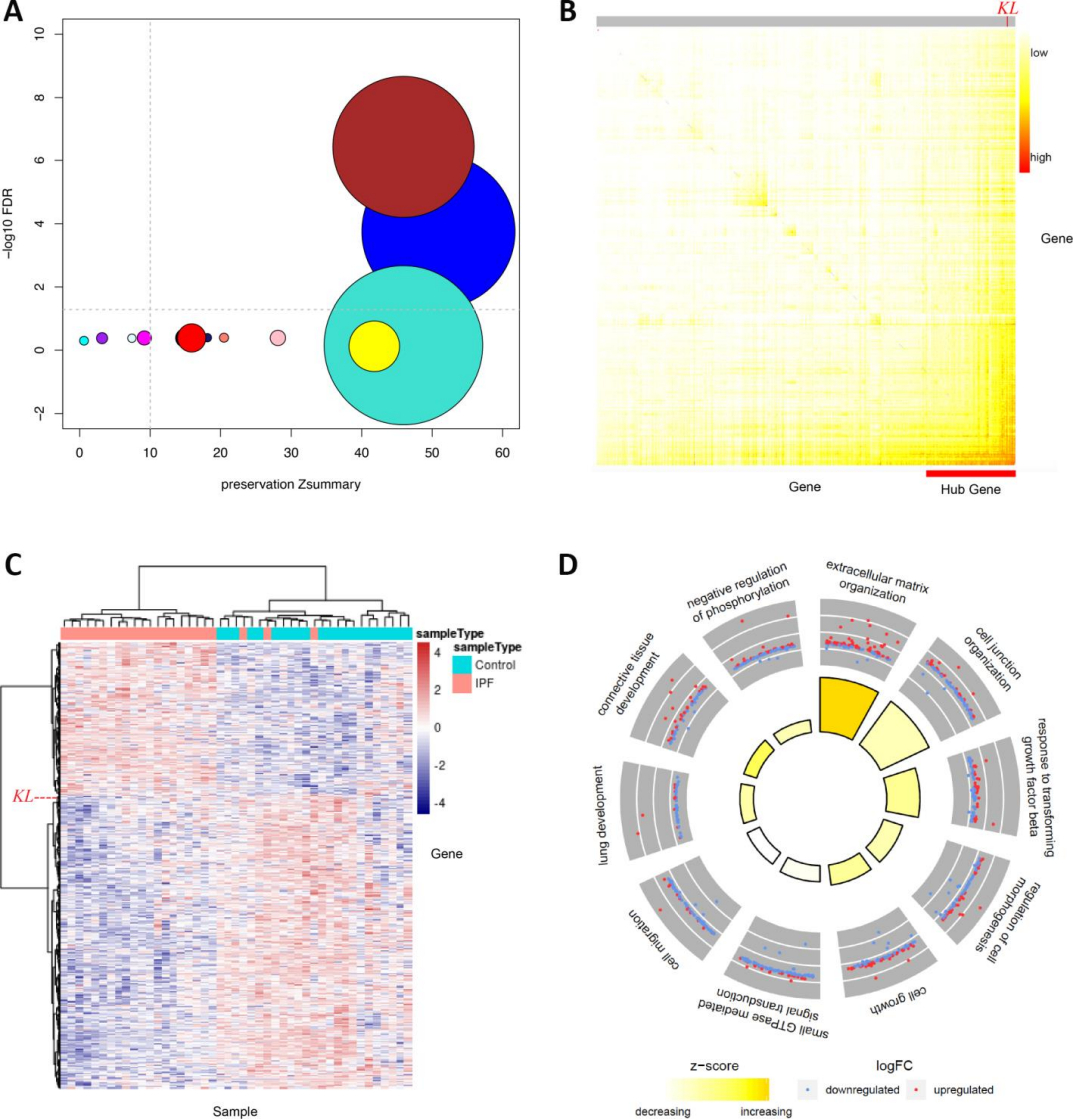
**WGCNA analysis identifies *KL* as the top IPF-relevant gene from the systems-level perspective.** (**A**) Gene network modules from IPF patients and normal controls are well preserved, two of which (BROWN and BLUE) are significantly associated with IPF. The x axis represents preservation statistics for the corresponding module, and y axis shows the correlation between each module and the clinic trait. The size of each circle is proportional to the number of genes in each module. The horizontal line indicates the threshold for significant association between module and trait, while the vertical line for module preservation. (**B**) Network heatmap plot exhibits connectivity between genes of BROWN module. Higher co-expression relationship is indicated by red colors. Hub genes are positioned at the left-bottom corner as they display high connectivity with most of the remaining of the genes. The position of *KL* is specified. (**C**) Heatmap shows relative expression of 3544 genes in BROWN module. The expression of *KL* is specified by a dashed line. (**D**) Circular visualization of the results of gene annotation analysis shows enriched biological processes of BROWN module. The outer circle is a scatter plot showing the log fold change of the assigned genes for each process. The inner circle is a bar plot whose height is proportional to the extent of enrichment for each process. Filled colors corresponds to a z-score that is a crude measure of how likely the biological process is to be decreased or increased.

**Figure 2:**
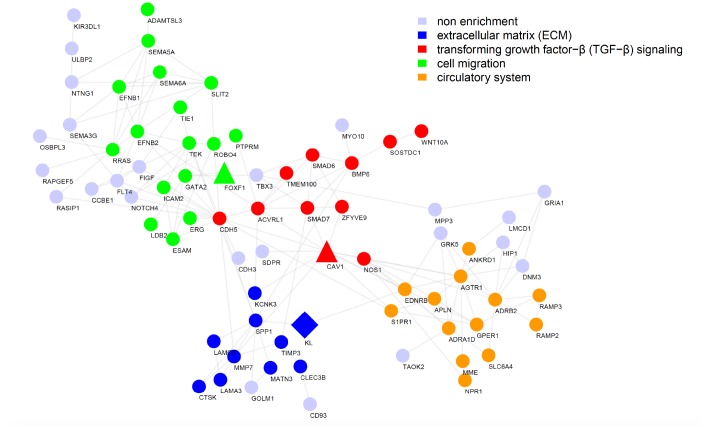
**Functional interaction network reveals the most likely pathways that could be regulated by *KL* in IPF.** Functional interaction network generated by the STRING database for the top 200 genes highly correlated with *KL* in BROWN module, with nodes representing genes and edges representing interactions. Disconnected genes were not shown.

The previous analysis focused on the detection of individual genes whose expression changes may be responsible for/responsive to IPF pathology [20]. It, however, would be more insightful to uncover disrupted functional networks enriched with malfunctioning genes. We thus performed WGCNA analysis and detected 16 modules with more than 200 genes, most of which had highly preserved network structures between conditions, implying there were no obvious perturbations in regulatory patterns (Figure 1A). Two modules, referred to as BROWN and BLUE, were significantly associated with IPF after multiple testing correction (FDR < 0.05), suggesting that the functional networks underlying these two modules play key roles in the development of IPF (Figure 1A). BROWN comprised 3544 genes, while BLUE comprised 3841 genes, collectively accounting for 30.4% (7385 out of 24297 in total) included in this analysis. Interestingly, BROWN network was dominated by a few highly connected genes (hubs), including *KL*, *ACVRL1*, *RAMP2*, and *LAMA3* (Figure 1B). These hub genes are likely key genes that regulate the expression of the module and thereby considered important for the phenotype of interest. Expression heatmap showed that IPF cases and controls could be well segregated by expression profiles of genes in BROWN (Figure 1C). Functional enrichment analysis related BROWN module to biological processes including cell migration, TGF-β signaling, and ECM, the latter two of which were more likely more active upon underexpression of *KL* (Figure 1C, 1D). Notably, STRINGDB network based on genes tightly correlated in expression with *KL* highlighted similar biological processes, lending credence to *KL*’s hub gene’s function in BROWN module (Figure 2). In summary, our integrative network analysis prioritizes *KL* as the top disease-relevant gene for further biological validation.

### *Kl* expression decreases in the murine pulmonary fibrosis model induced by bleomycin

**Figure 3:**
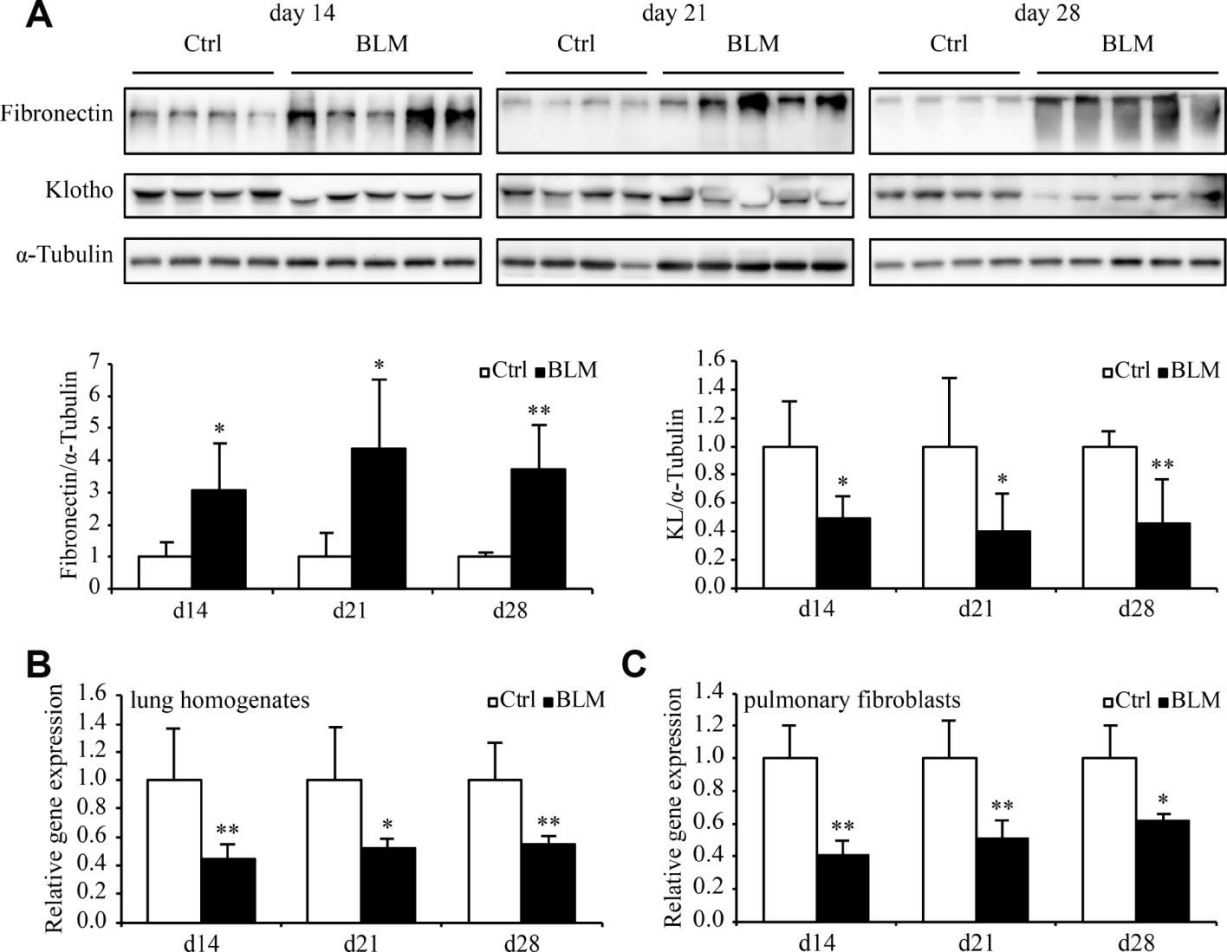
***Kl* expression decreases in the murine pulmonary fibrosis model induced by bleomycin.** Protein levels of fibronectin, KL, and α-Tubulin (**A**) were assessed by western blotting and mRNA levels of *Kl* were measured by qPCR (**B**) in the total lung lysates from mice 14, 21, and 28 days after intratracheally administering a single dose of PBS (Ctrl, white bars) or bleomycin (BLM, black bars). mRNA levels of *Kl* in the primary pulmonary fibroblasts (**C**) isolated from mice 14, 21, and 28 days after intratracheally administering a single dose of PBS (Ctrl, white bars) or bleomycin (BLM, black bars) were analyzed by qPCR. *P < 0.05 or **P < 0.01 *vs.* Ctrl. 5-7 animals per group.

We assessed *Kl* expression in mouse lungs and pulmonary fibroblasts during bleomycin-induced pulmonary fibrosis. Eight-week-old wild-type C57BL/6 mice were intratracheally administered a single dose of bleomycin. The body weights (Supplementary Figure 1A), both hematoxylin and eosin (H&E) and Masson-trichrome staining of lung tissues (Supplementary Figure 1B), and immunofluorescence staining of collagen I, fibronectin, and α-smooth muscle actin (α-SMA) in the lungs (Supplementary Figure 1C) all confirmed that bleomycin caused severe pulmonary fibrosis. We evaluated the progress of lung fibrosis 14, 21, and 28 days after bleomycin administration. Both the protein and mRNA levels of *Kl* decreased in every timepoint we examined (Figure 3A and 3B). Likewise, diminished *Kl* mRNA levels were observed in the primary pulmonary fibroblasts from mice 14, 21, and 28 days after exposure to bleomycin. Therefore, *Kl* is downregulated in both total lung lysates and isolated pulmonary fibroblasts in the murine pulmonary fibrosis model induced by bleomycin.

### *Kl* markedly ameliorates pulmonary fibrosis in an *ex vivo* model

**Figure 4:**
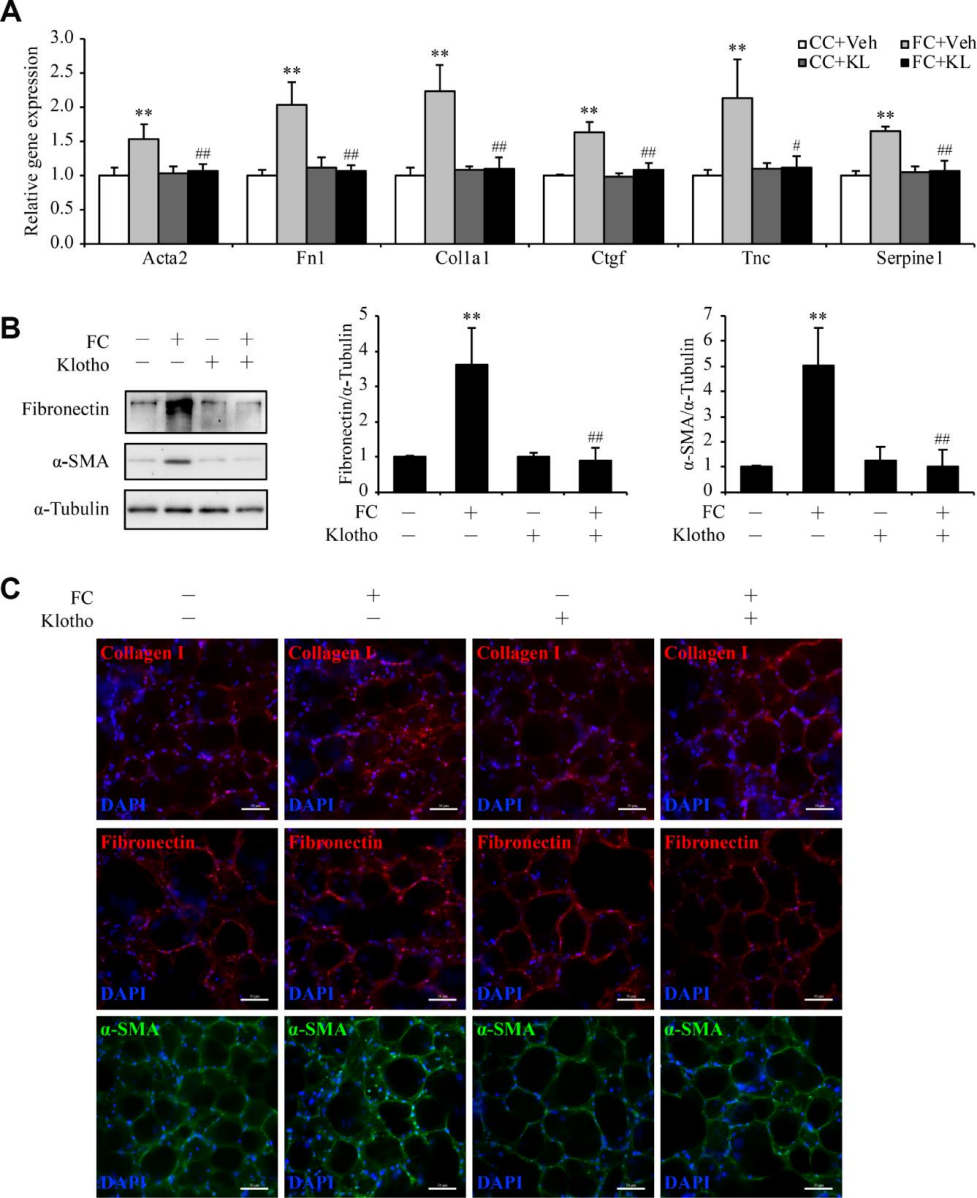
***Kl* markedly ameliorates pulmonary fibrosis in an *ex vivo* model.** Mouse PCLSs that were pre-incubated with vehicle (Vel) or mouse rKL for 24 h were randomized to be treated with control cocktail (CC) or fibrosis cocktail (FC) with or without KL for another 48 h. mRNA levels of *Acta2*, *Fn1*, *Col1a1*, *Ctgf*, *Tnc*, and *Serpine1* were assessed by qPCR (**A**), protein levels of fibronectin and α-SMA were measured by western blotting (**B**), and fibronectin, α-SMA, and collagen I were stained by immunofluorescence staining (**C**) in the mouse PCLSs from each group (CC+Veh, white bars; FC+Veh, light gray bars; CC+rKL, dark gray bars; FC+rKL, black bars). Scale bars = 50 μm. **P < 0.01 *vs.* CC+Veh. #*P* < 0.05 or ##*P* < 0.01 *vs.* FC+Veh.

Next, we established an *ex vivo* model of early-stage IPF by treating precision-cut lung slices (PCLSs) from wild type C57BL/6 mice with fibrotic cocktail (FC) which was composed of TGF-β, tumor necrosis factor-α (TNF-α), platelet-derived growth factor-AB (PDGF-AB), and lysophosphatidic acid (LPA) [21], and examined the potential anti-fibrotic effect of *Kl* in this model by adding mouse rKL into the medium. The upregulation of fibrotic genes including *Acta2*, *Fn1*, *Col1a1*, *Ctgf*, *Tnc*, and *Serpine1* stimulated by FC was significantly inhibited by rKL (Figure 4A). Meanwhile, compared to FC-incubated PCLSs, those that incubated with both FC and rKL displayed revered protein levels of α-SMA, fibronectin, and collagen I (Figure 4B and 4C). Thus, *Kl* exerted a protective effect against fibrosis in our *ex vivo* early pulmonary fibrosis model.

### *Kl* alleviates TGF-β-induced pulmonary fibroblasts activation, migration, and ECM production

**Figure 5:**
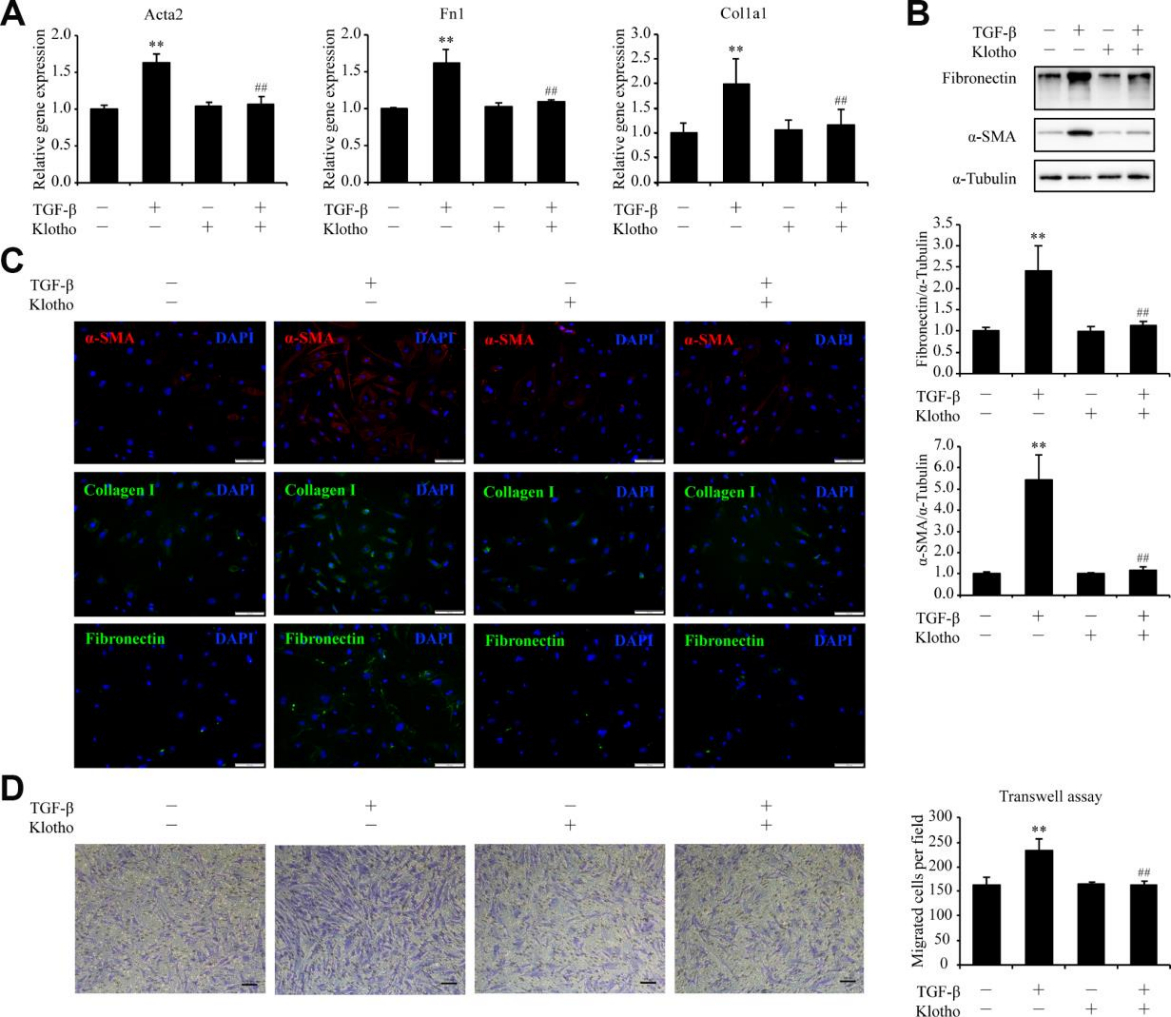
***Kl* markedly ameliorates pulmonary fibrosis in an *ex vivo* model.** Primary pulmonary fibroblasts isolated from wild type C57BL/6 mice were pre-incubated with or without mouse rKL. After 12 h, they were randomized to be incubated with or without TGF-β and KL for another 24 h when mRNA levels of *Acta2*, *Fn1*, and *Col1a1* were assessed by qPCR (**A**), protein levels of fibronectin, α-SMA, and α-Tubulin were examined by western blotting (**B**), fibronectin, α-SMA, and collagen I were stained by immunofluorescence staining (**C**), and migration of pulmonary fibroblasts were analyzed by transwell assay (**D**). Scale bars = 100 μm. **P < 0.01 *vs.* without TGF-β and without rKL. ##*P* < 0.01 *vs.* with TGF-β and without rKL.

The observations that *Kl* was significantly reduced in pulmonary fibroblasts during the development of pulmonary fibrosis in our study and that *KL* was predicted to modulate genes in TGF-β signaling led to the proposition that *KL* may antagonize TGF-β signaling to exert its protective effects in pulmonary fibroblasts. To test this hypothesis, we assessed activation, migration, and ECM production of isolated mouse pulmonary fibroblasts in response to TGF-β, a critical mediator of pulmonary fibrotic process. Both α-SMA (*Acta2*), the marker of myofibroblast and fibronectin (*Fn1*) as well as collagen I (*Col1a1*), the typical components of ECM, were upregulated by TGF-β. Mouse rKL supplementation was sufficient to reduce the increased expression of *Acta2*, *Fn1*, and *Col1a1* in TGF-β-treated pulmonary fibroblasts almost to the levels in control cells, as measured by qPCR (Figure 5A), western blot (Figure 5B), and immunofluorescence staining (Figure 5C). In cell migration assay, rKL was also found to inhibit the enhanced migration of pulmonary fibroblasts stimulated by TGF-β (Figure 5D). Collectively, these data suggest that *Kl* is able to attenuate pulmonary fibroblasts activation, migration, and ECM production during pulmonary fibrosis.

### *Foxf1* deletion reverses the inhibitory effect of *Kl* on pulmonary fibroblasts migration

**Figure 6:**
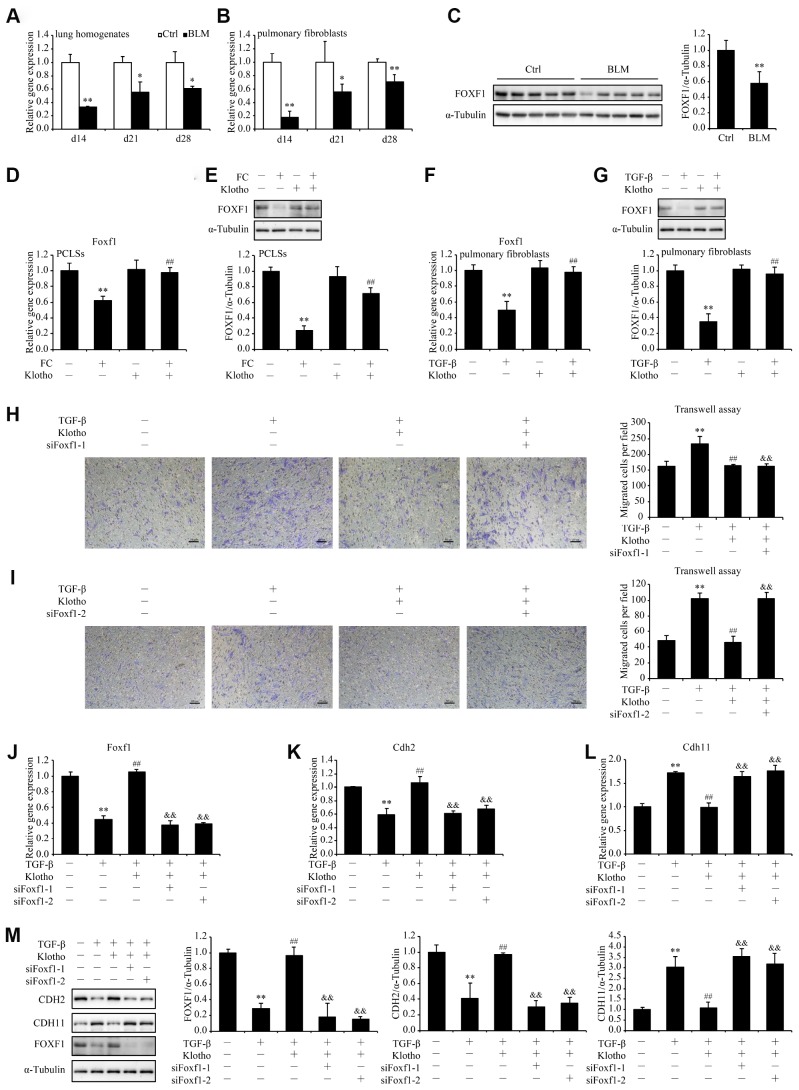
***Foxf1* deletion reverses the inhibitory effect of *Kl* on pulmonary fibroblasts migration.** mRNA levels of *Foxf1* were measured by qPCR in the total lung lysates (**A**) and isolated pulmonary fibroblasts (**B**) from mice 14, 21, and 28 days after intratracheally administering a single dose of PBS (Ctrl, white bars) or bleomycin (BLM, black bars). Protein levels of FOXF1 (**C**) were examined by western blotting in the total lung lysates from mice 21 days administering a single dose of PBS (Ctrl) or bleomycin (BLM). *P < 0.05 or **P < 0.01 *vs.* Ctrl. 5-7 animals per group. Mouse PCLSs pre-incubated with or without mouse rKL for 24 h were randomized to be treated with control cocktail (CC) or fibrosis cocktail (FC) with or without rKL for another 48 h, when mRNA (**D**) and protein (**E**) levels of *Foxf1* in the mouse PCLSs from each group were measured by qPCR and western blotting, respectively. **P < 0.01 *vs.* with CC and without rKL. ##*P* < 0.01 *vs.* with FC and without rKL. Primary pulmonary fibroblasts isolated from wild type C57BL/6 mice were pre-incubated with or without mouse rKL. After 12 h, they were randomized to be incubated with or without TGF-β and rKL for another 24 h when mRNA (**F**) and protein (**G**) levels of *Foxf1* were examined by qPCR and western blotting, respectively. **P < 0.01 *vs.* without TGF-β and without rKL. ##*P* < 0.01 *vs.* with TGF-β and without rKL. Primary pulmonary fibroblasts isolated from wild type C57BL/6 mice were transfected with control siRNA (siNC) or *Foxf1* siRNAs (siFoxf1-1 and -2). After siRNA transfection for 24 h, fibroblasts were pre-incubated with or without mouse rKL for 12 h, followed by treatment with or without TGF-β and rKL for another 24 h, when migration of pulmonary fibroblasts were analyzed by transwell assay (**H** and **I**, Scale bars = 100 μm), mRNA (**J**–**L**) and protein levels (**M**) of *Foxf1*, *Cdh2*, and *Cdh11* were measured by qPCR and western blotting, respectively. **P < 0.01 *vs.* siNC without TGF-β or rKL. ##*P* < 0.01 *vs.* siNC with TGF-β and without rKL. &&*P* < 0.01 *vs.* siNC with TGF-β and rKL.

Inspired by SRINGDB-based *KL*-centric network (Figure 2), we validated the role of *Foxf1* in cell migration. We observed significant decreases in the mRNA levels of *Foxf1* in both lung lysates and primary pulmonary fibroblasts from mice 14, 21, and 28 days after exposure to bleomycin (Figure 6A and 6B). The decreased protein levels of *Foxf1* were also confirmed by western blot (Figure 6C). Since *Foxf1* has been reported to inhibit pulmonary fibrosis by preventing CDH2 to CDH11 cadherin switch in myofibroblasts during pulmonary fibrosis [22], we proposed that *Kl* be very likely to repress the TGF-β-induced migration of pulmonary fibroblasts via enhancing the expression of *Foxf1*. As hypothesized, rKL supplementation restored *Foxf1* mRNA and protein levels in both PCLSs treated with FC (Figure 6D and 6E) and pulmonary fibroblasts incubated with TGF-β (Figure 6F and 6G). Next, endogenous *Foxf1* was silenced in pulmonary fibroblasts by siRNAs. The protective effect of rKL on pulmonary fibroblasts migration against TGF-β was abolished by *Foxf1* deletion (Figure 6H and 6I). Meanwhile, the expression of *Cdh2* and *Cdh11*, the reported targets regulated by *Foxf1* in pulmonary fibroblasts [22], also changed correspondingly (Figure 6J–6M), suggesting that the genes regulated by *Foxf1* could constitute an important mechanism by which *Kl* exerts its effect.

### The mitigation of TGF-β-induced pulmonary fibroblasts activation and ECM production by *Kl* is partially suspended after *Cav1* being silenced

**Figure 7:**
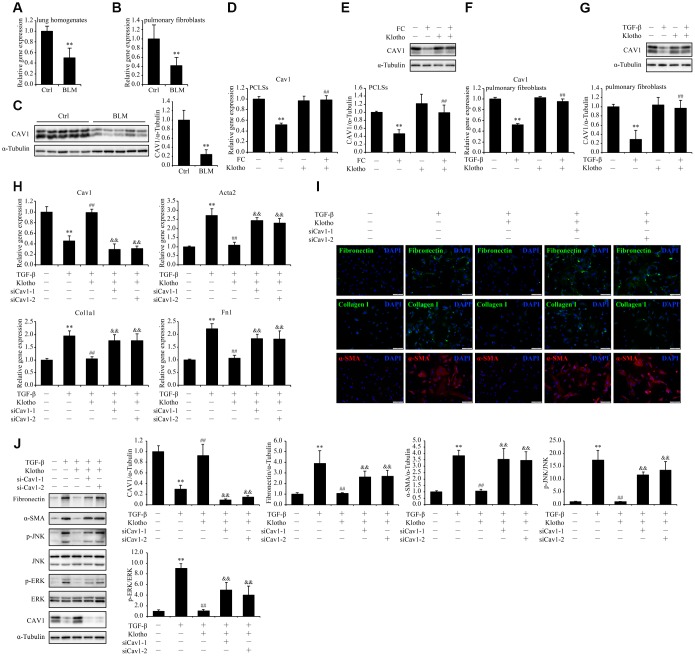
**The mitigation of TGF-β-induced pulmonary fibroblasts activation and ECM production by *Kl* is partially suspended after *Cav1* being silenced.** mRNA levels of *Cav1* were measured by qPCR in the total lung lysates (**A**) and isolated pulmonary fibroblasts (**B**) from mice 21 days after intratracheally administering a single dose of PBS (Ctrl) or bleomycin (BLM). Protein levels of CAV1 (**C**) were examined by western blotting in the total lung lysates from mice 21 days administering a single dose of PBS (Ctrl) or bleomycin (BLM). **P < 0.01 *vs.* Ctrl. 5-7 animals per group. Mouse PCLSs pre-incubated with or without mouse rKL for 24 h were randomized to be treated with control cocktail (CC) or fibrosis cocktail (FC) with or without rKL for another 48 h, when mRNA (**D**) and protein (**E**) levels of Cav1 in the mouse PCLSs from each group were measured by qPCR and western blotting, respectively. **P < 0.01 *vs.* CC without KL. ##*P* < 0.01 *vs.* FC without rKL. Primary pulmonary fibroblasts isolated from wild type C57BL/6 mice were pre-incubated with or without mouse rKL. After 12 h, they were randomized to be incubated with or without TGF-β and rKL for another 24 h when mRNA (**F**) and protein (**G**) levels of *Cav1* were examined by qPCR and western blotting, respectively. **P < 0.01 *vs.* without TGF-β or KL. ##*P* < 0.01 *vs.* with TGF-β and without rKL. Primary pulmonary fibroblasts isolated from wild type C57BL/6 mice were transfected with control siRNA (siNC) or *Cav1* siRNAs (siCav1-1 and -2). After siRNA transfection for 24 h, fibroblasts were pre-incubated with or without mouse rKL for 12 h, followed by treatment with or without TGF-β and rKL for another 24 h, when mRNA levels of *Cav1*, *Acta2*, *Fn1*, and *Col1a1* were assessed by qPCR (**H**), fibronectin, α-SMA, and collagen I were stained by immunofluorescence staining (**I**, Scale bars = 100 μm), and protein levels of fibronectin, α-SMA, p-JNK, JNK, p-ERK, ERK, CAV1 and α-Tubulin were examined by western blotting (**J**). **P < 0.01 *vs.* siNC without TGF-β or rKL. ##*P* < 0.01 *vs.* siNC with TGF-β and without rKL. &&*P* < 0.01 *vs.* siNC with TGF-β and rKL.

Likewise, the STRINGDB network drew our attention to *CAV1* in search of the mechanism underlying the inhibitory effect of *KL* on fibroblasts activation and ECM production in pulmonary fibroblasts. Encoding Caveolin-1 which is a principal component of caveolae, *Cav1* is capable of suppressing major profibrotic signaling pathways and antagonizing potentially profibrotic physiological events [23]. In accordance with past observations [24], we confirmed the downregulation of both *Cav1* mRNA and protein levels in total lung lysates as well as isolated pulmonary fibroblasts from bleomycin treated mice (Figure 7A–7C). Given the pivotal role of *Cav1* in fibroblasts activation and ECM regulation through ERK and JNK pathways [25], we postulated that *Kl* suppresses the TGF-β-induced fibroblasts activation and ECM production by restoring the expression of *Cav1*. As expected, both the mRNA and protein levels of *Cav1* were significantly reduced by FC and TGF-β in PCLSs and pulmonary fibroblasts, respectively, while rKL supplementation restored the expression of *Cav1* almost to the same levels of those in controls (Figure 7D–7G). Subsequently, we found that the amelioration of TGF-β-induced pulmonary fibroblasts activation and ECM production by rKL was partly suspended after *Cav1* had been depleted by siRNAs, with induction of both ERK and JNK phosphorylation at the same time (Figure 7H–7J). Taken together, these findings indicated that *Cav1* could mediate the inhibitory effect of *Kl* on TGF-β-induced pulmonary fibroblasts activation and ECM production.

## DISCUSSION

In the light of bioinformatic prediction and experimental validation, this study presented extensive characterization of the anti-fibrotic role of *KL* in pulmonary fibrosis, rendering it a promising target of therapeutic intervention for patients with IPF. Our transcriptomic network analysis identified *KL* as one of top candidate genes that drive the development of IPF. The subsequent experiments supported the “dry” findings by demonstrating that mouse rKL supplementation was sufficient to alleviate pulmonary fibrosis in an *ex vivo* model and to suppress the activation, migration, and ECM production induced by TGF-β in isolated mouse pulmonary fibroblasts. The experimentally validated functional dependencies between *KL* and predicted co-expressed genes in IPF, such as *FOXF1* and *CAV1*, deepened our comprehension of the molecular mechanisms underlying IPF etiology and *KL*’s anti-fibrotic effect.

Our observation that mouse rKL along was sufficient to antagonize fibrosis in pulmonary fibroblasts is inconsistent with a recent finding that FGF23 is required for human rKL to take anti-fibrotic effect [13]. This discrepancy is very likely to be attributed to the structural difference between the rKL proteins used in two studies, rather than the sequence difference between human and mouse KLs. The human rKL purchased by Barnes from PeproTech has only a calculated molecular weight of 58.6 kDa, while the mouse rKL used in our study was from R&D systems, with a predicted molecular weight of 109.5 kDa. As a corollary, the former only contains KL1 domain and the latter contains both KL1 and KL2 domains. Since the conformation created by the two extracellular KL domains (KL1, KL2) is indispensable to the interaction between KL and FGF23 and the following signaling [26], it is disputable that there is crosstalk between solely KL1 and FGF23, much less the claimed anti-fibrotic effect. Therefore, it is more plausible to attribute the protective effect against pulmonary fibrosis to KL containing both KL1 and KL2 domains. In their study, we noticed that both KL1 and FGF23 alone were able to increase only marginally the mRNA level of *KL* while the combination of FGF23 and KL1 led to a significant upregulation of *KL* mRNA level in pulmonary fibroblasts. Accordingly, we speculate that the expression of *KL* enhanced by FGF23 and KL1 is very likely to be responsible for the amelioration of TGF-β-induced fibrotic markers in pulmonary fibroblasts after KL1 and FGF23 co-administration. However, we cannot explain the exact mechanisms by which *KL* expression is upregulated by KL1 and FGF23 both separately and synergistically. Although mouse KL has about 80% homology with that of human [27], the sequence difference between human and mouse KL cannot be ignored. Thus, the anti-fibrotic effect of human rKL (with both KL1 and KL2 domains) should be confirmed in the future.

Not surprisingly, other hub genes identified in BROWN module appear to be involved in the pathology of IPF, implicitly supporting the proposed contribution of *KL* to IPF. *ACVRL1* encodes a type I cell-surface receptor for the TGF-β superfamily of ligands, which forms ACVRL1/SMAD1 axis and potentiates pulmonary fibroblasts activation [28]. *RAMP2* overexpression in myofibroblasts was reported to enhance survival and reduce pulmonary fibrosis in the bleomycin mouse model by sensitizing adrenomedullin signaling [29]. *LAMA3* is one of the few IPF risk genes reported so far [30]. Moreover, we found that the other IPF-associated module (BLUE) was overrepresented by cilium-activity-related genes, which is consistent with the emerging role of the primary cilium as a potential mediator of fibrosis pathogenesis [31]. The large quantity of genes (roughly 7000 in total) in these two modules may, at a minimum, partially explain the heterogeneous nature of etiology in IPF, while the small number of disease-associated modules is in line with the notion that many disease-causing genetic variants should cluster into key pathways that drive complex disease etiology.

Cautions should be taken in comprehension of enrichment result in Figure 1D. The direction of change predicted by GOplot is likely to be misleading when a biological process is dominated by genes without significant change, as the case for cell migration in our study. Only two genes (Supplementary Table 1 and Supplementary Table 2) showed large and significant fold change in the opposite direction between IPF and control. The up-regulated in IPF is PRSS3, a well-known oncogene that promote cell migration and cancer metastasis [32], while the down-regulated in IPF is MIR126, which functions as a tumor suppressor that inhibit cell migration [33]. Thus, changes in both genes support enhanced cell migration in IPF, in contrast to the prediction made by GOplot.

According to the STRINGDB network, co-expressed genes with *KL* were grouped into several functional categories including cell migration, TGF-β signaling, and ECM (Figure 2), revealing the most likely biological processes that could be regulated by *KL* in IPF, which was later confirmed by us *in vitro*. In the research of mechanisms, one co-expressed gene, *FOXF1*, which is critical for lung development and morphogenesis [34] was preferentially studied. Previously, Belkhir et al. reported that *Foxf1* expression was induced by the antifibrotic mediator prostaglandin E2 and repressed by TGF-β in pulmonary fibroblasts [35]. The vital role of *FOXF1* in the pathogenesis of IPF was later highlighted by the observation that loss of *Foxf1* increased migration of pulmonary fibroblasts via facilitating CDH2-CDH11 cadherin switch and thus aggravated bleomycin-induced pulmonary fibrosis in mice [22]. Consistent with these literatures, our results showed the significant downregulation of *Foxf1* in both fibrotic mice lungs and primary mouse pulmonary fibroblasts incubated with TGF-β and further demonstrated that rKL was sufficient to reverse the reduction of *Foxf1*, which subsequently attenuated TGF-β-induced migration of pulmonary fibroblasts through preventing CDH2-CDH11 cadherin switch. This protective effect of rKL was abolished by *Foxf1* deletion. However, little is known about the exact mechanism of *Foxf1* downregulation in pro-fibrotic environment. Additionally, we cannot tell whether the expression of *Foxf1* is directly regulated by *Kl* or indirectly via blocking the inhibitory effect of TGF-β for instance, which needs further exploration.

Intriguingly, similar functional dependency has been discovered between *KL* and another co-expressed gene, *CAV1*. As an integral membrane protein encoded by *CAV1*, Caveolin-1 which mainly composes the structure of caveola is implicated in numerous cellular processes, such as endocytosis, directional cell motility, and cell cycle regulation [36, 37]. Reduced CAV1 has long been observed in both IPF lungs [24, 25] and BLM-induced fibrotic mice lungs [24], which is consistent with our results. Recently, accumulated evidence has suggested its fibrosis suppressive potential in lung [23, 25, 38] and other tissues [23, 39]. Importantly, the decline of *Cav1* in pulmonary fibroblasts was reported to be associated with fibroblasts proliferation [40], activation, ECM production [25], and resistance to apoptosis [41], all of which are typical pathological characteristics of pulmonary fibroblasts during the development of pulmonary fibrosis [19, 42]. Oppositely, overexpression of *Cav1* is able to reverse these pathological phenotypes [25, 40, 41]. In accord with these studies, our results exhibited the repression of *Cav1* by FC and TGF-β in PCLSs and primary mouse pulmonary fibroblasts, respectively, which could be reversed by rKL. Although, it is yet unknown how rKL restores *Cav1* expression in our study, the mediatory role of CAV1 in the inhibitory of fibroblasts activation and ECM deposition by rKL was supported by the result that this protective effect was largely diminished with reactivated JNK and ERK signalings when *Cav1* was silenced. Additionally, it is noteworthy that *Cav1* is not exclusively expressed in fibroblasts. Previous researches uncovered its implication in apoptosis of lung epithelial cells [43], endothelial-mesenchymal transition (EndoMT) of pulmonary endothelial cells [44], dysregulated activation and recruitment of immune cells, and the resultant lung inflammation [45, 46] during pulmonary fibrosis. Whether the similar regulation of *Cav1* by *Kl* or the anti-fibrotic effect could take place in these cell types needs to be confirmed in the future.

In summary, this study revealed the decline of *Kl* expression in pulmonary fibroblasts as well as the protective effect of rKL on pulmonary fibroblasts in terms of activation, migration, and ECM production under pro-fibrotic conditions. Two co-expressed genes of *KL* in IPF lungs, *FOXF1* and *CAV1*, which could be restored by *KL*, were essential to the protective effect of *KL* on pulmonary fibroblasts against TGF-β. Our findings complement the existing mechanisms by which *KL* protects against fibrosis. Further *in vivo* studies on the anti-fibrotic role of *KL* in the lung and those that on the exact molecular mechanisms of *KL*-mediated actions in all kinds of cell types in and even out of the lung during the development of pulmonary fibrosis are badly needed to facilitate *KL*-based treatment strategy targeting pulmonary fibrosis, which is extremely promising.

## MATERIALS AND METHODS

### Bioinformatic analysis

We used Weighted Gene Co-expression Network Analysis (WGCNA) [47] to identify co-expressed gene modules from the RNA-seq data (log-transformed FPKM) of 23 samples with IPF and 22 control samples obtained from the LGRC website (https://www.lung-genomics.org/research/) [20], and subsequently test modules between IPF and control for differential expression (FDR<0.05). To assess if there was perturbation in genetic regulatory pattern in cases relative to controls, we capitalized on module preservation statistic Zsummary that defines the degree of preservation of network connectivity over conditions. In general, Zsummary > 10 implies hard evidence for module preservation. Hub genes, highly interconnected with other genes in a module, have been shown to be functionally significant at the system level [47]. To identify major contributors responsible for system-wide alteration, we searched for hub genes in differentially expressed modules. Enrichment analysis is performed by a R package (GOplot) [48], which offers an easy way to predict if certain biological process is more likely to be decreased or increased by simply dividing the difference between numbers of up-regulated and down-regulated genes by square root of total count of genes. To better illustrate the potential role of *KL* predicted by co-expression relationship, we leveraged the STRING database to construct a high-confidence functional network based on the top 200 co-expressed genes with *KL* from a disease-associated module (BROWN) with *KL* being one of its hub genes.

### Bleomycin-induced pulmonary fibrosis models

All animal experiments were approved by the Institutional Animal Care and Use Committee at Nanjing Medical University. Male C57BL/6 mice of 8 weeks were purchased from the National Resource Center for Mutant Mice Model Animal Research Center of Nanjing University. For bleomycin administration, mice were anaesthetized with avertin (Sigma Aldrich, St. Louis, MO, USA) followed by intratracheal instillation of bleomycin (HISUN PFIZER PHARMACEUTICALS CO.,LTD, Zhejiang, China) at a single dose of 1.5 U/Kg of body weight on day 0 in 50 μl of sterile saline.

### Primary pulmonary fibroblasts isolation, culture, and treatment

Primary pulmonary fibroblasts were isolated from unchallenged C57BL/6 mice, bleomycin-, and saline-treated mice as previously described [49]. Briefly, fresh mouse lung was cleared of blood by perfusion with ice-cold sterile PBS through the right ventricle and minced with a sterile scalpel blade into 1 mm^3^ fragments. Minced lung was suspended in 5 ml of digestion solution consisting of 5 mg/ml collagenase I (Sigma Aldrich) and 0.33 U/ml DNase I (Roche, Basel, Switzerland) in PBS and incubated with frequent agitation at 37 °C for 45 min. Cells were then filtered through a 70 μg strainer (Merck and Millipore, Darmstadt, Germany), centrifuged at 540 g for 5 min at 4 °C, and plated in tissue culture flasks in Dulbecco’s modified Eagle’s medium (DMEM) (Gibco, Burlington, ON, USA) with 15% fetal bovine serum (FBS) (Gibco), 100 μg/ml streptomycin, 100 U/ml penicillin, 0.25 μg/ml amphotericin B, and 10 mmol/l HEPES (Sigma Aldrich) at 37°C in a humidified 5% CO_2_ atmosphere. Cells were passaged after being harvested with trypsin-EDTA (Sigma Aldrich). Cells from passage 3 to 6 were used. For TGF-β treatment, 50 ng/ml recombinant klotho (R&D system, Minneapolis, MN, USA) was added to serum-free medium 12 h after serum withdrawal. After 12 h of KL pre-incubation, pulmonary fibroblasts were incubated with or without 5 ng/ml TGF-β (PeproTech, Rocky Hill, NJ, USA) for another 24 h.

### Mouse precision-cut lung slices (PCLSs) preparation, culture, and treatment

PCLSs were generated as previously described [50]. Briefly, warm, low gelling temperature agarose (2% by weight, Sigma) in sterile DMEM/Ham’s F12 (Gibco) was infiltrated into mouse lung through trachea. After the trachea being ligated with thread to retain the agarose inside the lung, the whole lung was excised and cooled on ice for 10 min to allow gelling of the agarose. Then the separated lobes were cut with a vibratome (VT1000 S, Leica, Buffalo Grove, IL, USA) to a thickness of 300 μm. The PCLSs were cultivated in DMEM/Ham’s F12 (Gibco) with 0.1% FBS (Gibco), 100 μg/ml streptomycin, 100 U/ml penicillin, 0.25 μg/ml amphotericin B, and 10 mmol/l HEPES (Sigma Aldrich) at 37°C in a humidified 5% CO_2_ atmosphere. For control cocktail (CC) or fibrosis cocktail (FC) treatment, 50 ng/ml rKL was added to serum-free medium 12 h after serum withdrawal. After 24 h of klotho pre-incubation, mouse PCLSs were incubated with CC or FC for another 48 h. Both the CC and FC were prepared as previously described [21] (28314802). In brief, FC contains 5 ng/ml TGF-β (PeproTech), 5 μM PDGF-AB (PeproTech), 10 ng/ml TNF-α (PeproTech), and 5 μM LPA (Cayman Chemical, Ann Arbor, MI, USA).

### Transient transfection

Small interfering RNAs (siRNAs) were designed and synthesized by Ribobio (Guangzhou, Guangdong, China). For transient transfection, Lipofectamine RNAiMAX reagent (Invitrogen, Grand Island, NY, USA) was mixed with siRNAs according to the manufacturer’s protocol as perviously described [51].

### Western blot analysis

Western blotting was performed as previously described [51]. Individual immunoblots were probed with rabbit anti-α-KL pAb (ABclonal, Wuhan, Hubei, China) diluted 1:1000, mouse anti-α-SMA mAb (Sigma Aldrich) diluted 1:2000; rabbit anti-Fibronectin pAb (Abcam, New Territories, HK, China) diluted 1:1000, goat anti-FOXF1 pAb (R&D Systems) diluted 1:1000, rabbit anti-CDH2 pAb (ABclonal) diluted 1:1000, rabbit anti-CDH11 pAb (ABclonal) diluted 1:1000, rabbit anti-CAV1 (Proteintech, Wuhan, Hubei, China) diluted 1:2000, rabbit anti-JNK pAb (Cell Signaling Technology, Danvers, MA, USA) diluted 1:1000, rabbit anti-p-JNK mAb (Cell Signaling Technology) diluted 1:1000, rabbit anti-ERK1/2 mAb (Cell Signaling Technology) diluted 1:1000, rabbit anti-p-ERK1/2 mAb (Cell Signaling Technology) diluted 1:1000, mouse anti-α-Tubulin mAb (Sigma Aldrich) diluted 1:4000 in 2.5% (wt/vol.) non-fat dried milk in Tris-buffered saline with Tween-20 (TBST) buffer.

### RNA extraction and RT-PCR assay

Total RNA was extracted from cells and lungs using Trizol reagent (Invitrogen). Mouse PCLSs were washed twice with PBS and snap frozen in liquid nitrogen. Afterwards, the FastPure Cell/Tissue Total RNA Isolation Kit (Vazyme, Nanjing, Jiangsu, China) was used with modifications of the protocol provided by the manufacturer to isolate total RNA from mouse PCLSs. RNA was reverse-transcribed into cDNA with PrimeScript™ RT Master Mix (Takara, Shiga, Japan). Quantification RT-PCR was performed using ChamQ^TM^ Universal SYBR qPCR Master Mix (Vazyme) and Step One Plus^TM^ Real-Time PCR System (Applied Biosystems, Foster City, CA, USA). *Arpppo* was used as internal standards for mRNAs. The primers used are as follows (5’-3’): mouse *Fn1* (forward: ACCAGGTTGATGATACTTCC, reverse: TCTCCTCCACAGCATAGATAG); mouse *Acta2* (forward: CAGGGAGTAATGGTTGGA, reverse: AGTGTCGGATGCTCTTCA); mouse *Col1a1* (forward: GATGTCCTATGGCTATGATGAA, reverse: ACCCATTGGACCTGAACC); mouse *Kl* (forward: TATTGATGGCGACTACCC, reverse: GGCGGAACTTCATGTTAG); mouse *Foxf1* (forward: TGTCTGGCAGCATCTCCA, reverse: TCCTCCGCCTGTTGTATG); mouse *Cdh2* (forward: AGTCTTACCGAAGGATGTG, reverse: CCTGGGTTTCTTTGTCTT); mouse *Cdh11* (forward: GTCTCCTCATGGCTTTGC, reverse: CTTTAGATGCCGCTTCAC); mouse *Cav1* (forward: ACGAGGTGACTGAGAAGC, reverse: AGACAACAAGCGGTAAAA); *Arpppo* (forward: GAAACTGCTGCCTCACATCCG, reverse: GCTGGCACAGTGACCTCACACG).

### Immunofluorescence staining

Immunofluorescence staining on tissue sections, mouse PCLSs, and primary pulmonary fibroblasts was performed as described previously [50, 52]. In brief, immunofluorescent staining on frozen sections (5 μm), PCLSs, and primary pulmonary fibroblasts was performed using primary antibodies to α-SMA (1:200, Sigma Aldrich), Collagen I (1:200, Abcam), Fibronectin (1:200, Abcam) and associated fluorescein (FITC)- and Cy3-conjugated secondary antibodies (Jackson ImmunoResearch, West Grove, PA, USA) per manufacturer instructions. Stained sections were imaged using OLYMPUS automated fluorescence microscope BX63 (OLYMPUS, Shinjuku, Tokyo, Japan) and stained mouse PCLSs were imaged under ZEISS laser scanning confocal microscope LSM5 (ZEISS, Oberkochen, Germany).

### Cell migration assays

To assess the migrative ability of pulmonary fibroblast, 20,000 fibroblasts were seeded in 400 μl of serum-free medium with or without mouse rKL supplementation onto an 8 μm 24-well hanging insert (Merck and Millipore) and cell migration was performed in the presence of 10% FBS complete medium with or without TGF-β1. After 48 h, media were removed and the polycarbonate filters with the migrated cells were fixed stained with crystal violet (Beyotiome, Shanghai, China). The migrated cells of each sample were counted in ten randomly selected fields.

### Statistical analysis

At least three independent experiments were performed. Comparisons were performed using the Student’s t test between two groups or ANOVA in multiple groups. Results were presented as means ± SEM. A value of *P* < 0.05 was considered statistically significant.

## Supplementary Material

Supplementary Figure 1

Supplementary Table 1

Supplementary Table 2
