# Supplementary material for: Klotho antagonizes pulmonary fibrosis through suppressing pulmonary fibroblasts activation, migration, and extracellular matrix production: a therapeutic implication for idiopathic pulmonary fibrosis
Source: Aging (Albany NY). 2020 Apr 3;12(7):5812–31. doi: 10.18632/aging.102978 (PMC7185122; doi:10.18632/aging.102978)
Supplement: Supplementary Figure 1 [file aging-12-102978-s002..pdf]

SUPPLEMENTARY FIGURE

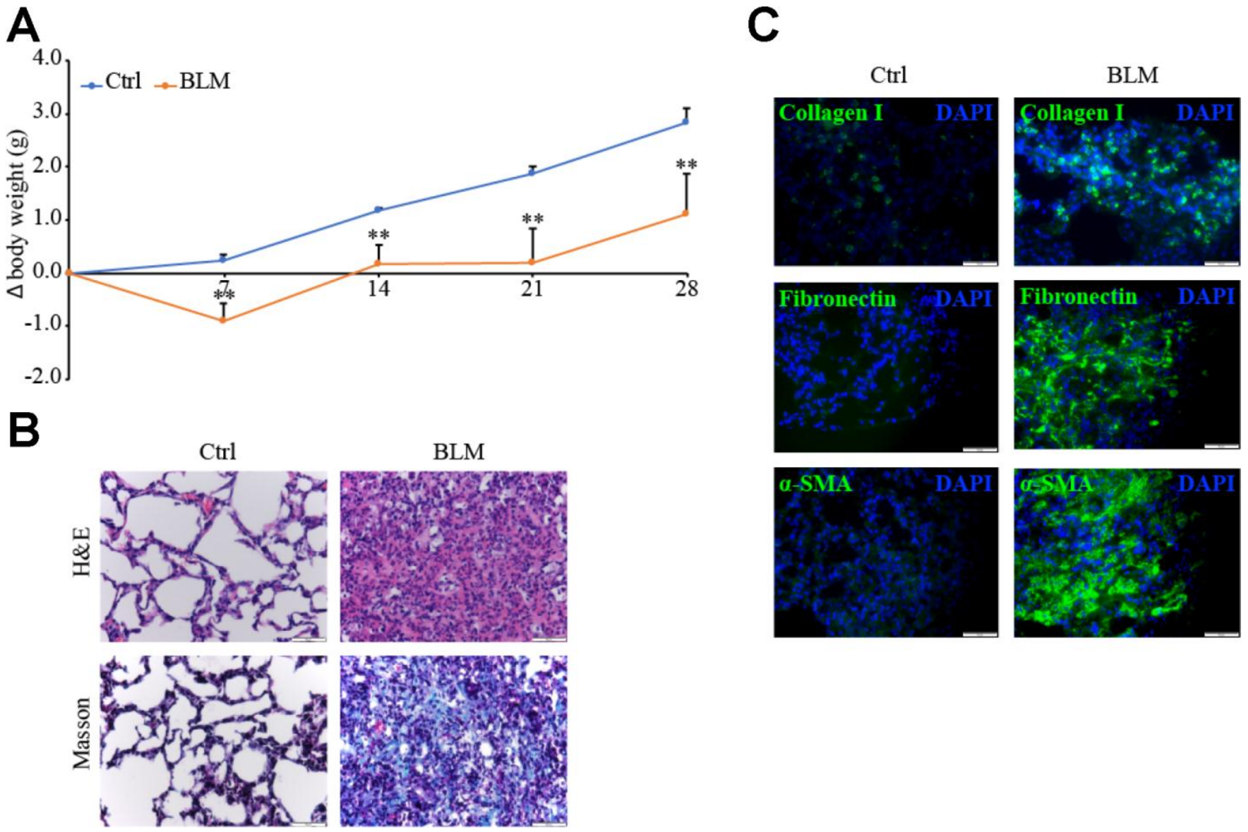

Supplementary Figure 1. Validation of bleomycin induced pulmonary fibrosis in mice. Eight-week-old mice were intratracheally administered a single dose of PBS (Ctrl) or bleomycin (BLM). Body weight (BW) was monitored daily and is depicted as change in grams relative to baseline (0 day) (**A**). The lungs were harvested and subjected to H&E staining, Masson trichrome staining (**B**), and immunofluorescence staining of fibronectin, α-SMA, and collagen I (**C**). Scale bars = 50 μm. **$P < 0.01$ *vs.* Ctrl. 5-7 animals per group.
