# Supplementary material for: Klotho antagonizes pulmonary fibrosis through suppressing pulmonary fibroblasts activation, migration, and extracellular matrix production: a therapeutic implication for idiopathic pulmonary fibrosis
Source: Aging (Albany NY). 2020 Apr 3;12(7):5812–31. doi: 10.18632/aging.102978 (PMC7185122; doi:10.18632/aging.102978)
Supplement: Supplementary Table 2 [file aging-12-102978-s001..pdf]

Supplementary Table 2. All genes in each enrichment term showed in Figure 1D.

GO_ID	Description	geneID
GO:0030198	extracellular matrix organization	“ITGAL,ITGA3,PHLDB1,IBSP,VCAN,CDH1,TNC,GPM6B,LAMC3,LAMA3,COL11A1,COL17A1,FSCN1,COL5A3,COL4A4,COL19A1,COL16A1,MMP2,CCDC80,CMA1,TGFB2,TLL2,MADCAM1,MMP11,PDGFB,CTSG,MMP9,FERMT1,TIMP1,RGCC,FOXF1,SERPINE1,ENG,SPOCK2,SH3PXD2A,ICAM2,COL1A1,VWF,SMOC2,LAMA4,COL7A1,TNR,NID1,MMP8,TTR,CCN2,SPP1,TGFBI,FMOD,RECK,PRDX4,TCF15,KDR,PXDN,COL5A1,RAMP2,MATN3,POSTN,ITGA7,FOXF2,SULF1,MMP7,MMP13,ITGA11,CYP1B1,ADAMTS14,FBLN5,COL6A1,COL6A2,APP,CCN1,ITGA10,DPT,CTSK,SFRP2,NOTCH1,SERPINH1,MMP3,WNT3A,JAM2,MMP14,NPHS1,PDPN,COL6A3,IHH,FBLN2,MELTF,EGFLAM,COL1A2,JAM3,FBN1,MFAP4,MMP10,GREM1,KLK5,COL3A1,COL4A3,PTK2,COL22A1,FGG,COL8A2,LAMB2,EFEMP2,HPSE2,SH3PXD2B,GAS6,OLFML2A,COL14A1,SULF2,MMP1,COL27A1,PDGFA,COL13A1,ADAMTSL2,COL11A2,COL5A2,COL15A1,PRSS1,ATXN1L”
GO:0034330	cell junction organization	“NR1H4,PRKCH,VCL,CDH1,EPHA3,DSG2,GPM6B,ARHGAP6,LAMA3,PKP2,HDAC7,CDH3,LIMCH1,COL17A1,RHOA,SDK2,CDH19,ACTN1,LIMS2,MPP5,PVR,FERMT2,FSCN1,ACTN2,TNS1,OPHN1,COL16A1,ADD1,TGFB2,NRP1,PPM1F,SMAD7,PARD6A,TJP1,NUMBL,LIM2,CAV1,SRF,VEGFA,CDH6,EPB41L5,TGFB3,TEK,PARD6B,MACF1,KDR,FLNC,NECTIN2,ACTN4,RAMP2,NUMB,TLN1,ACVRL1,CDH13,IQSEC1,PARD3,TAOK2,THY1,PRKCA,RHOC,MMP14,RUNX1,ACE,FBLIM1,CADM3,FZD5,CLDN1,DLC1,MICALL2,ILK,GREM1,MAPRE2,DAPK3,FAM107A,PTK2,GJB1,CTNND2,NLGN2,CDH2,PEAK1,HEG1,UGT8,CD151,RCC2,CDH5,F2R,GJC1,CADM1,CLDN5,EFNA5,KRT14,GJA4,APOD,PRTN3,S100A10,OCLN,MYO1C,CTNND1”
GO:0071559	response to transforming growth factor beta	“CREBBP,ITGA3,FYN,FUT8,HSPA5,LTBP1,RHOA,PRKCZ,HYAL2,TGFBR3,FERMT2,EDN1,PXN,LTBP4,TGFB2,BAMBI,FERMT1,SMAD7,MXRA5,PARD6A,CAV1,WNT2,ENG,BMPR1A,COL1A1,GCNT2,WNT5A,EPB41L5,ACVR1,FNDC4,LTBP2,TGFB3,SMAD9,APAF1,FMOD,CITED1,GDF5,ID1,CDKN1C,GDF15,POSTN,WNT10A,TGFBRAP1,LRRC32,SMAD6,CILP,ACVRL1,PML,MYOCD,PRDM16,CGN,LEFTY2,STAR,CDKN2B,PARD3,ANKRD1,KLF10,NODAL,ZFYVE9,SKI,ZYX,CLDN1,CLEC3B,HPGD,CITED2,COL1A2,FBN1,MAPK7,COL3A1,LDLRAD4,PTK2,FOS,JUN,CDH5,PENK,CAV3,CLDN5,ADAMTSL2,BMPR2,UBA52”
GO:0022604	regulation of cell morphogenesis	“SEMA3F,HECW1,PLXND1,ARHGAP44,SEMA3G,FYN,SEMA3B,NEDD4L,RHOA,SYT1,FERMT2,PLXNA2,MAP2,CARMIL1,SEMA5B,GSK3B,ULK2,CASS4,SEMA6A,UNC13D,BAMBI,NRP1,PALM,TRIOBP,COCH,NFATC4,FGD1,FBXO31,SYT17,RAB11A,ANKRD27,MAG,PTN,CXCL12,C1QBP,CCL7,RASAL1,NEDD9,BVES,SRF,VEGFA,SEMA5A,WNT5A,TTL,TNR,MPL,APOA1,MYL12B,SPP1,BCL11A,ARAP3,CPNE5,ID1,RHOJ,FGD3,MACF1,KDR,ACTN4,OLFM1,DLG4,WASF3,MYH10,POSTN,EPHB2,LRP4,ADGRB3,CAPRIN1,ITGA7,ENPP2,TRPC6,SEMA6D,SSH2,WTIP,CRABP2,SEMA6C,RHOB,LIMD1,SLIT2,MYO10,CDC42EP2,TAOK2,EPS8,THY1,WNT3A,FGD5,RHOC,ARMCX5-GPRASP2,S100B,BRSK1,CHRNB2,FMNL3,ZSWIM5,FBLIM1,PDPN,CDC42EP3,NKX6-1,MELTF,DLC1,RET,ILK,NDEL1,DAPK3,SEMA6B,SHOX2,EFNA1,PTK2,SEMA3E,CDH2,FGG,DSCAM,CCL11,RND1,FZD4,BDNF,RCC2,MYADM,CCL13,AMIGO1,EPHB3,CNTN2,EFNA5,ROBO2,PRKN,DCC,S100A10,DNM3,ARC,BMPR2,PLXNA4,SHANK3”
GO:0016049	cell growth	“SEMA3F,CD38,ADIPOR2,SEMA3G,SEMA3B,IGF1,PRDM11,VCL,NEDD4L,RHOA,PRKCZ,SYT1,HYAL2,ENO1,MAP2,EDN1,SEMA5B,GSK3B,ULK2,LTBP4,SEMA6A,TGFB2,NRP1,BDKRB1,CSNK2A1,WFDC1,SYT17,RAB11A,SPG11,CCN4,MAG,CXCL12,TMEM97,RASAL1,SRF,DNPH1,VEGFA,SEMA5A,HBEGF,BCL6,WNT5A,HYAL1,TTL,EPB41L5,IGFBP5,TNR,NRP2,CCN2,SPP1,BCL11A,SOCS2,ADRA1A,CDKN2C,CPNE5,MACF1,EIF2AK4,INO80,CDKN2D,BST2,OLFM1,PAK4,ARHGEF11,DCLK1,POSTN,BTG1,PSRC1,RERG,SERPINE2,MAP2K5,SEMA6D,PLCE1,CLSTN3,ACVRL1,FBLN5,PML,MYOCD,TP53,RPTOR,IGFBP4,APP,CCN1,CRABP2,SEMA6C,TMEM108,AGTR1,SLIT2,SFRP2,IGFBP3,LAMTOR1,TAOK2,WNT3A,MMP14,FRZB,IGFBP7,NKX6-1,SOX17,AVPR1A,ILK,NDEL1,GREM1,SERTAD3,SEMA6B,IGFBP6,GNG4,FAM107A,NPR1,SEMA3E,DSCAM,SLC25A33,LAMB2,NET1,SFN,BDNF,SERTAD2,AGTR2,CAV3,EFNA5,PRKN,NRG3,DCC,FAM122A,DCUN1D3,BMPR2,FGFR1OP,SIPA1,MEG3,PLXNA4”
GO:0051056	regulation of small GTPase	“ITGA3,ARHGAP44,CYTH3,IGF1,RALBP1,ARHGAP31,ARHGAP6,GDI2,CDON,RHOA,RHOBTB1,OPHN1,TGFB2,SYDE2,NRP1,FGD1,PPP2CB,ARHGEF10,SYDE1,PIK3R2,TNFAIP1,ARHGEF

	mediated signal transduction	17,RASAL1,BCL6,RALGPS2,MFN2,SIPA1L2,APOA1,TRIM67,ARAP3,ADRA1A,ARHGAP40,TRIP10,RHOJ,FGD3,F2RL3,ARHGEF6,STARD8,EPO,ARHGEF9,RAP1GAP2,ARHGEF11,STARD13,EPHB2,ARHGEF4,SPRY2,DAB2IP,ARRB1,ARHGAP20,ARHGAP29,PLCE1,ARHGAP24,ITPKB,RHOB,GPR17,IQSEC1,SLIT2,NOTCH1,EPS8,FARP1,OBSCN,FGD5,RHOC,PSD3,VAV2,ARHGAP25,ARHGEF3,DLC1,ARHGEF40,MAPRE2,COL3A1,SCAI,NET1,ARHGAP1,GPR4,F2R,SPRY4,SRGAP1,KANK2,ARHGEF15,GPR20,ARHGAP19,SIPA1”
GO:0090130	cell migration	“PLXND1,ITGA3,PRSS3,ADGRA2,FLT4,HDAC7,RHOA,VASH1,EDN1,PXN,TGFB2,NRP1,PPM1F,PDGFB,MMP9,CD40,ANGPT4,FERMT1,RGCC,FOXF1,RAB11A,GPI,HDAC5,IFNG,SMOC2,SRF,VEGFA,SEMA5A,HBEGF,WNT5A,HYAL1,EPB41L5,APOA1,NRP2,TEK,PKN1,NR4A1,PLCG1,EFNB2,ID1,RHOJ,MACF1,KDR,DLL4,SERPINF1,STARD13,KLF4,DAB2IP,ENPP2,MAP2K5,CYP1B1,ANXA3,ACVRL1,CDH13,DUSP10,RHOB,IQSEC1,SLIT2,NOTCH1,VEGFC,ANGPT1,PRKCA,CXCL13,PAXIP1,STC1,PKN3,JCAD,AMOTL1,GREM1,MAPRE2,ATOH8,EFNA1,PTK2,MMRN2,PTPRM,CLEC14A,JUN,GATA2,AGTR2,ADGRB1,PRKX,PRKD1,EPHB4,KANK2,MAP3K3,MIR126,BMPR2,EMP2,TNFSF12,INSL3”
GO:0030324	lung development	“ITGA3,FLT4,TNC,FSTL3,SREBF1,PGR,TBX5,PHEX,MAPK3,FOXF1,PTN,WNT2,HOXA5,BMPR1A,PTK7,SRF,VEGFA,WNT5A,IGFBP5,EPAS1,ERRFI1,HSD11B1,CCN2,TCF21,TGFB3,TBX4,SPDEF,ID1,THRA,KLF2,CHI3L1,SPRY2,TNS3,HS6ST1,STRA6,MYOCD,GATA6,ABCA12,GPC3,RSPO2,NOTCH1,NODAL,MMP14,SELENON,PDPN,VANGL2,PKDCC,HHIP,HEG1,PDGFA,ADAMTSL2,BMPR2,ATXN1L”
GO:0061448	connective tissue development	“NR1H4,COL11A1,HYAL2,EVC,ACAT1,EDN1,OXCT1,XBP1,PDGFB,COCH,ZNF516,TIMP1,MAPK3,HOXA5,BMPR1A,FBXW4,COL1A1,ZBTB16,SRF,WNT5A,COL7A1,HYAL1,EFEMP1,MEF2D,CCN2,TGFBI,EGR1,GDF5,THRA,COL5A1,RARA,MATN3,CHI3L1,ADAMTS7,SULF1,MMP13,ACVRL1,COL6A1,COL6A2,CARM1,CCN1,CTSK,WNT9A,SFRP2,RSPO2,SLC25A25,NOTCH1,SERPINH1,BMP6,PAXIP1,STC1,PTH1R,PKDCC,FRZB,NPPC,COL6A3,IHH,OSR2,GREM1,SCARA3,SHOX2,WNT10B,ID4,CD34,LEP,SH3PXD2B,PLAAT3,GPR4,CREB3L2,RFLNB,AMER1,COL14A1,SULF2,COL27A1,ANXA6,BMPR2,COL11A2”
GO:0042326	negative regulation of phosphorylation	“PRKAR2B,PAX6,GPRC5A,LMO3,IPO5,PPP2R5A,RHOA,PRKCZ,HYAL2,SIRT2,NCK2,TRIB2,RPS6KA6,BAX,SEMA6A,GADD45B,PPM1F,FBXO7,TIMP3,BDKRB1,SMAD7,PARD6A,PIK3R2,CAV1,AMBP,ENG,PPIF,STK38,H2AFY,CBLB,ERRFI1,DUSP1,TWIST1,CDKN2C,PKN1,CDKN2D,CHRNA10,CDKN1C,CBFA2T3,MLLT1,GMFG,RGN,SH3BP5,INPP5K,EPHB2,MICAL1,NIBAN1,SPRY2,KLF4,DAB2IP,ARRB1,SORL1,SMAD6,DUSP6,WARS,MYOCD,RPTOR,CBLC,DUSP10,CTDSP1,CTDSPL,SLIT2,SFRP2,IGFBP3,CDKN2B,PARD3,DRD2,LATS2,GPD1L,HHEX,THY1,ANGPT1,LRRK1,UCHL1,SAMSN1,PLPP3,CAMK2N1,ATF3,SPINK1,GPER1,PRDX3,NDRG2,ILK,GREM1,BDKRB2,LDLRAD4,PKIG,RGS14,EFNA1,PKIA,TRIB1,CTDSP2,SFN,JUN,PER1,MYADM,ADIPOQ,CAV3,DUSP8,PRKN,INKA1,IRS2,SPRY4,PRKAR1B,ADARB1,INKA2,SPRED2,TRIM27,FGFR1OP”
